# Endocrine Therapy-Resistant Breast Cancer Cells Are More Sensitive to Ceramide Kinase Inhibition and Elevated Ceramide Levels Than Therapy-Sensitive Breast Cancer Cells

**DOI:** 10.3390/cancers14102380

**Published:** 2022-05-12

**Authors:** Purab Pal, Alec Millner, Svetlana E. Semina, Rosemary J. Huggins, Logan Running, Diana S. Aga, Debra A. Tonetti, Rachel Schiff, Geoffrey L. Greene, G. Ekin Atilla-Gokcumen, Jonna Frasor

**Affiliations:** 1 Department of Physiology and Biophysics, College of Medicine, University of Illinois at Chicago, Chicago, IL 60612, USA; palpurab@uic.edu (P.P.); semina@uic.edu (S.E.S.); 2 Department of Chemistry, University at Buffalo, The State University of New York (SUNY), Buffalo, NY 14260, USA; alecmill@buffalo.edu (A.M.); loganrun@buffalo.edu (L.R.); dianaaga@buffalo.edu (D.S.A.); 3 Ben May Department for Cancer Research, University of Chicago, Chicago, IL 60637, USA; rhuggins@uchicago.edu (R.J.H.); ggreene@uchicago.edu (G.L.G.); 4 Department of Pharmaceutical Sciences, College of Pharmacy, University of Illinois at Chicago, Chicago, IL 60612, USA; dtonetti@uic.edu; 5 Lester and Sue Smith Breast Center, Baylor College of Medicine, Houston, TX 77030, USA; rschiff@bcm.edu

**Keywords:** ceramide, ceramide kinase, sphingolipid profiling, endocrine therapy resistance, breast cancer, ceramide-1-phosphate, tamoxifen resistance, ceramide-1-phosphate

## Abstract

**Simple Summary:**

Endocrine therapy (ET) resistance is a major problem in estrogen receptor-positive breast cancer patients. Since there have been few lipidomic studies in ET resistance and sphingolipids are heavily implicated in multidrug-resistant and chemotherapy-resistant cancers, we aimed to investigate the sphingolipidome of tamoxifen-resistant breast cancer cells in search of a unique sphingolipid profile that can potentially be exploited therapeutically. We found that ET-resistant breast cancer cells maintain a lower level of ceramides for their survival. In order to achieve this, they are dependent on ceramide kinase (CERK), the activity of which helps maintain low endogenous ceramide levels, therefore promoting tamoxifen-resistant cell survival. Targeting CERK can therefore represent an opportunity to target therapy-resistant breast tumors and improve the patient outcome for women with ET-resistant disease.

**Abstract:**

ET resistance is a critical problem for estrogen receptor-positive (ER+) breast cancer. In this study, we have investigated how alterations in sphingolipids promote cell survival in ET-resistant breast cancer. We have performed LC-MS-based targeted sphingolipidomics of tamoxifen-sensitive and -resistant MCF-7 breast cancer cell lines. Follow-up studies included treatments of cell lines and patient-derived xenograft organoids (PDxO) with small molecule inhibitors; cytometric analyses to measure cell death, proliferation, and apoptosis; siRNA-mediated knockdown; RT-qPCR and Western blot for gene and protein expression; targeted lipid analysis; and lipid addback experiments. We found that tamoxifen-resistant cells have lower levels of ceramides and hexosylceramides compared to their tamoxifen-sensitive counterpart. Upon perturbing the sphingolipid pathway with small molecule inhibitors of key enzymes, we identified that CERK is essential for tamoxifen-resistant breast cancer cell survival, as well as a fulvestrant-resistant PDxO. CERK inhibition induces ceramide-mediated cell death in tamoxifen-resistant cells. Ceramide-1-phosphate (C1P) partially reverses CERK inhibition-induced cell death in tamoxifen-resistant cells, likely through lowering endogenous ceramide levels. Our findings suggest that ET-resistant breast cancer cells maintain lower ceramide levels as an essential pro-survival mechanism. Consequently, ET-resistant breast cancer models have a unique dependence on CERK as its activity can inhibit de novo ceramide production.

## 1. Introduction

Representing about 15% of all cancer incidence in women in the US [1], breast cancer is one of the most common cancers in middle-aged and older women. About 75% of breast tumors are positive for estrogen receptor-α (ER+). Even though the hormone receptor-negative subtypes are more lethal and aggressive, ER+ breast cancer accounts for the majority of cancer-related deaths [2,3]. ER+ breast cancer patients are commonly prescribed endocrine therapy (ET), i.e., aromatase inhibitors (AI) or anti-estrogens such as tamoxifen or fulvestrant. Although most women initially respond to ET, 30–50% of women will experience relapse while on treatment or after finishing standard 5-year treatment, which indicates that ET resistance may exist de novo or develop as a consequence of extended ET [4,5,6]. Many recurring tumors tend to retain ER but no longer respond to ET [7]. Several mechanisms of ET resistance have been defined, including (i) alteration in ER signaling (for example, altered co-activator vs. co-repressor binding) [8,9] (ii) increased crosstalk between ER and other growth pathways (such as ER-HER2 crosstalk) [10,11,12], and (iii) constitutively activating mutations in *ESR1* [13,14].

Progress in our understanding of these resistance mechanisms has been made with extensive genomic, proteomic, and transcriptomic profiling studies [15,16,17,18]; however, the role of lipids in mediating ET resistance is poorly understood. Although some lipidomic profiling studies have been reported in breast cancer comparing cancerous and normal breast tissue [19,20,21,22,23,24] and one study in recurrent versus non-recurrent hormone receptor-negative breast cancer [25], to our knowledge, lipidomic profiling studies have not been performed in ET resistance.

One family of lipids that are closely involved in both chemotherapy resistance and multidrug resistance are sphingolipids. Sphingolipids are bioactive lipids involved in cell survival and death [26,27,28]. The sphingolipidome consists of a highly complex network of various molecular species. The bioactive sphingolipids include ceramides, sphingosines, ceramide-1-phosphate (C1P), and sphingosine-1-phosphate (S1P) and can interact with several targets, i.e., enzymes such as kinases, phosphatases, and lipases, or membrane receptors to exert distinct cellular actions [29]. The actions of these bioactive lipids are associated with numerous cellular processes such as cell migration, adhesion, survival, apoptosis, senescence, and inflammation [29]. Interestingly, sphingolipids are also implicated in insulin resistance [30], immune cell functions [31], and epigenetic regulation [32] in various conditions and diseases.

Evidence from the literature suggests that drug-resistant cancer cells need to regulate pro-apoptotic sphingolipid levels, i.e., ceramide levels, for survival [33,34]. In the 1990s, multidrug-resistant cervical cancer cells were reported to have elevated glycosylated ceramides, suggesting a higher ceramide turnover [35]. Since then, several studies in different cancers have established a connection between ceramides and drug resistance [36,37,38]. Interestingly, tamoxifen has been reported to affect sphingolipids in a few breast cancer studies. Tamoxifen treatment inhibited acid ceramidase in an ER-independent manner [39], and tamoxifen treatment in 4T1, MCF-7, and MDA-MB-231 cells increased the level of several species of ceramides associated with phosphorylation of JNK, and cleavage of caspase-3 and PARP [40]. In another study, tamoxifen induced S1P production and increased cell migration in an ESR1-splice variant (ERα36) breast cancer cell line [39]. While these findings implicate the regulation of sphingolipids in tamoxifen action, the role of sphingolipids in endocrine therapy resistance is largely unclear.

Therefore, in order to systematically investigate the global sphingolipidomic changes in ET-resistant breast cancer, we performed targeted profiling of the sphingolipidome of tamoxifen-sensitive and -resistant breast cancer cell lines and found that tamoxifen-resistant cells maintain lower levels of ceramides and hexosylceramides, suggesting an altered regulation for sphingolipid production and/or metabolism in tamoxifen resistance. Asking whether this alternative sphingolipidome exposes unique vulnerabilities that can be exploited to treat tamoxifen-resistant breast cancers, we found that tamoxifen-resistant cells are uniquely dependent on CERK. The product of CERK, C1P, can inhibit de novo ceramide production, an important consequence that specifically promotes ET-resistant breast cancer cell survival. This unique dependence on CERK can potentially be leveraged therapeutically to target ET-resistant breast tumors and improve outcomes for women with ET-resistant disease.

## 2. Materials and Methods

Materials: Lipid standards used in LC-MS analysis were purchased from Avantipolar Lipids: C16:0 ceramide-1-phosphate (#860533), C12:0 ceramide-1-phosphate (#860531), C8:0 ceramide-1-phosphate (#860532), C17 ceramide (#860517), C17 sphingomyelin (#860585), and C17 glucosylceramide (#860569). LC-MS columns and guard cartridges were purchased from Phenomenex (Torrance, CA, USA). NVP-231 (#13858), C8 ceramide-1-phosphate (d18:1/8:0) (#62547), and C8 ceramide (d18:1/8:0) (#62540) were purchased from Cayman Chemical Company (MI, USA). siCERK (Ambion s34929) and Silencer^TM^ negative control (siNEG) (Thermo Fisher Scientific, Waltham, MA, USA) were purchased from Sigma. 4-hydroxytamoxifen (4-OHT) was purchased from Sigma (#H7904). CERK (Rabbit) primary antibody was purchased from MyBiosource (MBS7047905), and β-actin (Mouse) primary antibody was purchased from Sigma (#A5441). Goat anti-rabbit (#31460) and anti-mouse (#31430) HRP conjugated secondary antibodies were purchased from Invitrogen (Waltham, MA, USA).

Cell culture and treatments: MCF-7, MCF-7-HER2, and BT474 cell lines were generously provided by Dr. Rachel Schiff (Baylor) and MCF-7-TAM1 and T47D cells were generously provided by Dr. Debra Tonetti (UIC). All cell lines were maintained in their standard growth conditions as previously described [41,42,43,44]. Cell line authentication using short tandem repeat (STR) and mycoplasma contamination testing were performed routinely for every cell line used.

For exogenous lipid treatment to cultured cells, we used C8-ceramide and C8-C1P to ensure intracellular delivery because of better solubility but similar bioactivity compared to their C16-counterparts [45,46].

Patient-derived xenograft organoid (PDxO) culture and treatment: HCI-011 and HCI-011-FR PDxOs were kindly provided by Dr. Alana Welm (University of Utah Huntsman Cancer Institute). All PDxOs were cultured and maintained as previously described [43]. For NVP-231 treatment, 10 µL of Matrigel-PDxO suspension (~50 PDxO/well) was pipetted into each well of a 48-well plate, creating a small dome. The plate was incubated at 37 °C for 10 min and then treatments were added to each well. Plates were analyzed every 12-h using the Incucyte S3 Organoid module to measure total organoid area per µm^2^.

Extraction of ceramides, sphingomyelins, and hexosylceramides: For initial sphingolipidomic analysis, extraction of sphingolipids, except for from ceramide-1-phosphate, was performed as described previously [47]. The samples were normalized based on protein content. The chloroform used for resuspension was spiked with C17:0-ceramide, C17:0-glucosylceramide, and C17:0-sphingomyelin for use as internal standards.

LC-MS analysis of ceramides, sphingomyelins, and hexosylceramides: LC-MS analysis was performed as described previously [47]. Briefly, data acquisition was performed with an Agilent 1260 HPLC coupled to an Agilent 6530 Accurate-Mass Quadrupole Time-of-Flight mass spectrometer. Ceramides were analyzed in negative mode [M-H]^−^; and sphingomyelins [M]^+^ and hexosylceramides [M-OH]^+^ were analyzed in positive mode.

Ceramide-1-phosphate extraction and analysis: An alternate extraction procedure was performed for ceramide-1-phosphate as described previously [48]. The samples were normalized based on their cell number and reconstituted in methanol. The methanol used for reconstitution was spiked with C17:0-ceramide, C8:0-ceramide-1-phosphate, C17:0-glucosylceramide, and C17:0 sphingomyelin for use as internal standards. LC-MS/MS was performed with an Agilent 1200 liquid chromatography tower coupled to a Thermo Scientific TSQ Quantum Ultra mass spectrometer [48].

C16:0 ceramide-1-phosphate has a precursor *m*/*z* of 618 with product *m*/*z* of 264 and 600, which are observed at a ratio of ~2:1, respectively, in a standard. In extract from different cell lines, interfering peaks with similar *m*/*z* to precursor and product ions are distinguished from C16:0 ceramide-1-phosphate by considering the proper ratio of product ions. It is important to note that LC separations of C16:0 ceramide-1-phosphate were sensitive to solvent composition and column performance. Solvents were prepared fresh for each analysis. All integrations were based on ~2:1 ratio of *m*/*z* = 264:600 and the retention time of the internal standards analyzed in an LC-MS run. Drifts in retention time of +/−1 min were deemed acceptable for all samples given the correct ratio of fragment ions. Injections of blank solvent were used after every sample to avoid carry-over. C16-C1P was the only C1P species detected in extracts from all three cell lines.

Extraction of ceramides, sphingomyelins, and hexosylceramides from NVP-231-treated samples and their analyses: Sphingolipids from these samples were extracted similar to how we describe in “Ceramide-1-phosphate extraction”. The analysis of these species is completed similar to how we describe in “LC-MS analysis of ceramides, sphingomyelins and hexosylceramides”.

Cell death analysis: Cell confluency and cell death were analyzed with a Celigo Imaging Cytometer (Nexcelom Bioscience, Lawrence, MA, USA), as we have previously described [49]. Briefly, cells were treated with Hoechst 33,432 (Life Technologies, Carlsbad, CA, USA) and propidium iodide (PI) (final concentration: 1 µg/mL) for 30 min and incubated for 30 min at 37 °C. PI-positive cells and total cells were then detected in the red and blue fluorescence channels, respectively. The percentage of dead cells was determined as the number of dead cells (positive red fluorescence signal) over the total number of cells (positive blue fluorescence signal) per well.

Apoptosis assay: Apoptosis assays were performed using ViaStain^TM^ No-Wash Annexin V-FITC kit (Nexcelom, CSK-V0007-1) following the manufacturer’s protocol. Apoptotic cells were measured by counting cells that are positive for annexin-V (green fluorescence channel) and propidium iodide (red fluorescence channel) normalized to total cells measured by DAPI (blue fluorescence channel) in a Celigo cytometer.

CERK knockdown: Cells were transfected with siRNA targeting CERK (Ambion, s34929 validated) or a non-targeting control (siNeg) (Thermo Fisher Scientific, Waltham, MA, USA) using DharmaFECT 1 (Dharmacon, Lafayette, CO, USA). Transient transfection was performed with a final concentration of 20 nM of siRNA in 4% DharmaFECT in OptiMEM (Gibco, Waltham, MA, USA). Media was changed to regular media after 24-h, and Hoechst-PI endpoint assay for cell death was performed at 72-h post-transfection.

RNA isolation and RT-qPCR: Total RNA isolation with Trizol (Ambion, Austin, TX, USA), cDNA synthesis, and RT-qPCR were performed as previously described with 36B4 and GAPDH as housekeeping controls [50]. Primer sequences are available upon request.

Western blot: Whole-cell extracts were prepared using M-PER reagent (Thermo Scientific, 78503 Waltham, MA, USA). Proteins were denatured and then separated by Novex^TM^ WedgeWell^TM^ 4–12% Tris-Glycine gel (Invitrogen, XP04120) and transferred to nitrocellulose membrane (Thermo Scientific, 88018). The membrane was blocked with 5% non-fat dry milk in 1% TBST and incubated overnight with respective primary antibodies [dilutions: 1:1500 of anti-CERK (rabbit) and 1:5000 of anti-β-actin (mouse) in blocking buffer] at 4 °C. Membranes were then washed and incubated with HRP-conjugated-secondary antibodies. The signal was visualized using Chemidoc MP (Bio-Rad laboratories, Hercules, CA, USA) with the Pierce Supersignal West Pico Chemiluminescent substrate (Thermo Scientific).

Analysis of CERK expression in human breast tumors: Briefly, the association between the mRNA expression level of CERK and relapse-free (RFS) or overall survival (OS) was analyzed using the KMplotter database (http://kmplot.com/analysis/ (accessed on 28 April 2022) in ER+ patients that received exclusively tamoxifen (*n* = 662 for RFS and *n* = 104 for OS) [51]. The Kaplan–Meier survival plots and log-rank *p*-values were obtained using KMplotter’s analysis algorithm.

Statistical analyses: All data are represented as mean +/− SEM from at least 3 independent samples. Statistical analyses were performed using GraphPad Prism v9.0. Student’s *t*-test and one-way or two-way ANOVA were used where appropriate. All *p*-values were two-sided and statistically significant change was considered *p* < 0.05.

## 3. Results

### 3.1. Results

#### 3.1.1. Tamoxifen-Resistant Cells Have an Altered Sphingolipidome and Rely on Ceramide Kinase (CERK) for Survival

**Figure 1:**
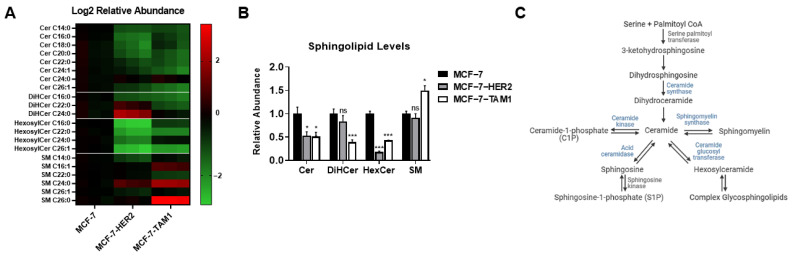
Targeted analysis of the sphingolipidome reveals decreased ceramides and hexosyl-ceramides in tamoxifen-resistant cells. (**A**) Abundance of different sphingolipid species in tamoxifen-resistant MCF-7-HER2 and MCF-7-TAM1 cells relative to tamoxifen-sensitive parental MCF-7 cells was determined by LC-MS. Relative abundances in log2 scale are presented as a heatmap. Relative abundances were calculated by dividing the abundance of a certain sphingolipid species by the average abundance of that species in MCF-7 cells. (**B**) Relative abundance of total ceramides (Cer), dihydroceramides (DiHCer), hexosylceramides (HexCer), and sphingomyelins (SM) in each tamoxifen-resistant cell line were calculated by dividing the abundance of a certain sphingolipid species by the average abundance of that species in MCF-7 cells and are presented as fold changes in a bar plot. (**C**) Simplified version of the human sphingolipid pathway is presented as a schematic diagram. Key enzymes in the pathway are shown in blue. ns: not significant, * *p* < 0.05, *** *p* < 0.001.

To probe for changes in sphingolipids associated with tamoxifen resistance, we performed a sphingolipidomic analysis of tamoxifen-sensitive parental MCF-7 cells and two of its tamoxifen-resistant derivatives, MCF-7-HER2, which was developed by HER2 overexpression [41] and MCF-7-TAM1, which was developed by long-term exposure to 4OHT [42]. We found that ceramide and hexosylceramide levels were reduced by more than 50% in both tamoxifen-resistant cell lines compared to parental MCF-7 cells (Figure 1A,B). Additionally, MCF-7-TAM1 cells had lower levels of dihydroceramides, and higher sphingomyelin levels compared to MCF-7 parental cells, but these changes were not observed in MCF-7-HER2 cells. These findings suggest that tamoxifen-resistant cells may have regulatory mechanisms to maintain lower levels of ceramides and hexosylceramides. Since the mammalian sphingolipidome consists of a complex interplay of several bioactive sphingolipid metabolites (Figure 1C), we asked whether these regulatory mechanisms can be exploited to target tamoxifen-resistant cell survival. To address this, we attempted to perturb the sphingolipid pathway with different inhibitors of key enzymes; however, most inhibitors either showed no effect on survival or no apparent difference in responses between tamoxifen-sensitive and -resistant cells (data not shown).

**Figure 2:**
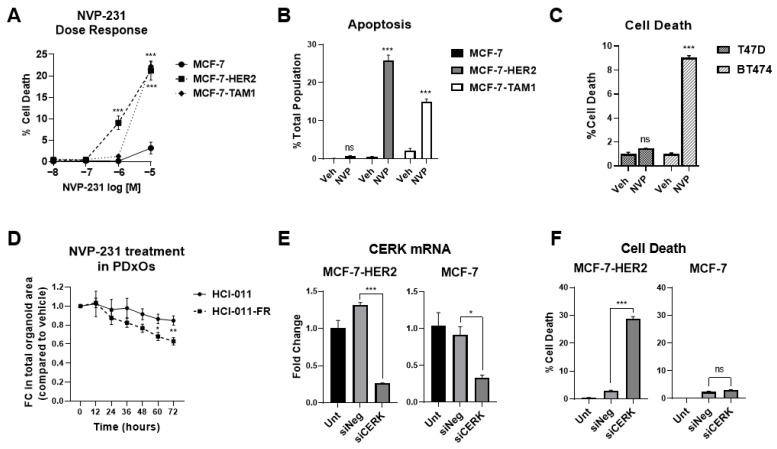
Endocrine therapy-resistant cells rely on CERK for survival. (**A**) MCF-7, MCF-7-HER2, and MCF-7-TAM1 cells were treated with increasing doses of NVP-231 for 48-h. Cell death was assessed by Hoechst-PI staining. (**B**) Annexin-V-positive and PI-positive cells were analyzed following a 48-h treatment of 1 µM and 10 µM NVP-231 to assess apoptosis. The percentage of apoptotic cells were calculated as the number of Annexin-V and PI-positive cells times 100 over total number of cells, as assessed by Hoechst counterstain. (**C**) T47D and BT474 cells were treated with 10 µM NVP-231 for 48-h. Cell death was assessed by Hoechst-PI staining. (**D**) Fulvestrant-sensitive (HCI-011) and -resistant (HCI-011-FR) PDxOs were treated with 10 µM NVP-231 for 72-h. Organoid growth was analyzed by measuring total organoid area. Data points are represented as fold changes in total organoid area by NVP-231 related to vehicle at each time point. (**E**,**F**) CERK was knocked down by a validated siRNA in MCF-7-HER2 and MCF-7 cells. An untransfected control and a non-specific siNeg-transfected control were included for each cell line. Cells were harvested after 72-h of transient transfection. RT-qPCR for CERK mRNA was performed to validate knockdown (**E**) and Hoechst-PI endpoint assay was performed to estimate cell death (**F**). ns: not significant, * *p* < 0.05, *** *p* < 0.001.

In contrast, tamoxifen-sensitive and tamoxifen-resistant cell lines displayed differential sensitivity to NVP-231, a potent and selective inhibitor of mammalian CERK [52]. We conducted cell viability and apoptosis studies with NVP-231 and found that it selectively reduces viability and induces apoptotic cell death in tamoxifen-resistant cells (MCF-7-HER2 and MCF-7-TAM1) to a higher magnitude than in tamoxifen sensitive parental MCF-7 cells (Figure 2A,B). We further validated our findings in another tamoxifen-sensitive (T47D) and a relatively tamoxifen-resistant (BT474) breast cancer cell line, where NVP-231 induced significant cell death in BT474 cells but not in T47D cells (Figure 2C), confirming that tamoxifen-resistant breast cancer cells are more sensitive to CERK inhibition.

To extend our observation to other models of ET-resistant breast cancer, we used the ER+ fulvestrant-sensitive (HCI-011) patient-derived xenograft organoid (PDxO) model, and its fulvestrant-resistant counterpart (HCI-011-FR), which was derived by allowing a fulvestrant responsive xenograft tumor to recur over a long-term fulvestrant treatment [53]. NVP-231 treatment inhibited the growth of HCI-011-FR significantly more than HCI-011 (Figure 2D), further supporting our finding that ET-resistant breast cancer models are more sensitive to CERK inhibition than ET-sensitive models.

We next validated that NVP-231 action is mediated by CERK using an siRNA approach. We found knockdown of CERK (Figure 2E) induced a significantly higher level of cell death compared to the non-targeting (siNeg) control in MCF-7-HER2 cells but not in the parental MCF-7 cells (Figure 2F).

To probe whether CERK inhibition directly affects the response to tamoxifen in tamoxifen-resistant models, we checked if CERK inhibition alters sensitivity to tamoxifen in MCF-7-HER2 cells. We observed that 4-hydroxytamoxifen (4-OHT) has no effect on MCF-7-HER2 cell survival, either alone or in combination with NVP-231, suggesting that CERK inhibition does not re-sensitize tamoxifen-resistant cells to tamoxifen and that the effect of CERK inhibition is likely to be independent of ER function (Supplemental Figure S1).

#### 3.1.2. Differences in CERK Expression and Activity Does Not Explain Increased Sensitivity of Tamoxifen-Resistant Cells to CERK Inhibition

**Figure 3:**
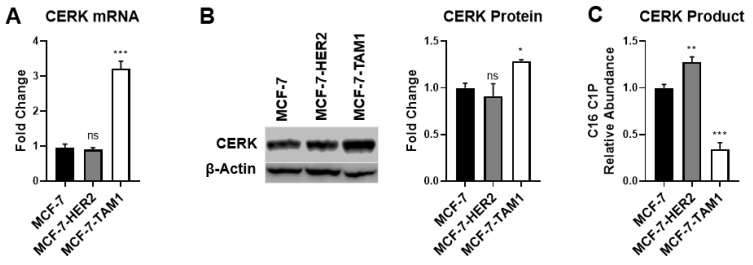
Tamoxifen-resistant cells do not have different CERK expression and/or product levels. (**A**) Total RNA was extracted and CERK mRNA was measured from MCF-7, MCF-7-HER2, and MCF-7-TAM1 cells by RT-qPCR. Fold change for CERK mRNA was calculated relative to CERK expression in MCF-7 cells. (**B**) Total protein was extracted and CERK protein levels were measured by Western blot. CERK protein expression was normalized to β-actin and was quantified as fold change relative to normalized CERK protein expression in MCF-7 cells. The uncropped Western blots have been shown in Figure S4. (**C**) C16-C1P level was measured in three cell lines by LC-MS/MS and relative levels of C16-C1P in the tamoxifen-resistant cell lines were quantified as fold changes relative to the C16-C1P level in MCF-7 cells. ns: not significant, * *p* < 0.05, ** *p* < 0.01, *** *p* < 0.001.

To understand why tamoxifen-resistant cells may be more sensitive to CERK inhibition, we first examined CERK expression. We observed that CERK mRNA and protein levels were elevated in MCF-7-TAM1 cells relative to MCF-7 cells, but CERK levels in MCF-7-HER2 cells were not different (Figure 3A,B), suggesting that the level of CERK expression likely does not explain differential sensitivity to CERK inhibition in tamoxifen-resistant cells. We also examined CERK expression in ER+ breast tumors treated with tamoxifen and found no association with relapse-free or overall survival in patients (Supplemental Figure S2). Since CERK expression appears not to be predictive of either response to CERK inhibition or patient outcome, we next investigated differences in CERK activity in tamoxifen-sensitive and -resistant cell lines. To do this, we measured intracellular C16:0 ceramide-1-phosphate (C16-C1P) levels as an indication of differences in the product of CERK enzyme activity. We note that C16-C1P is the only C1P species that we could detect in the cell lysates. We found that MCF-7-HER2 cells have a higher level of C16-C1P compared to MCF-7 cells, whereas the C16-C1P level in MCF-7-TAM1 cells was lower than MCF-7 cells (Figure 3C). These findings suggest that there are no consistent differences among cell lines in CERK expression or the level of its product (C1P) that explains the differential sensitivity of tamoxifen-resistant cells to CERK inhibition.

**Figure 4:**
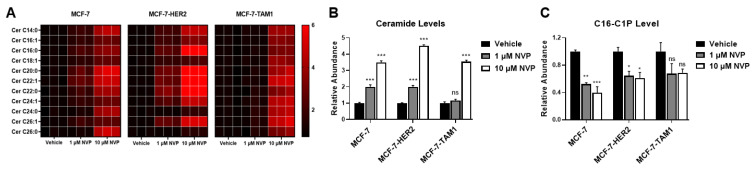
CERK inhibition induces similar changes in ceramides and C1P in all cell lines. (**A**) MCF-7, MCF-7-HER2, and MCF-7-TAM1 cells were treated with 1 µM and 10 µM NVP-231 for 48-h. Abundance of individual ceramide species in all cell lines was then measured relative to a vehicle-treated (DMSO) control for each cell line and presented as a heatmap. (**B**) Relative abundance of ceramides was calculated by dividing the abundance of ceramides in the NVP-treated groups by the average abundance of ceramides in vehicle-treated groups and data is presented as fold changes in a bar plot. (**C**) C16-C1P levels were assessed following 1 µM and 10 µM NVP-231 treatment for 48-h in all three cell lines. Data are represented as fold changes relative to C16-C1P levels in vehicle-treated control for each cell line. ns: not significant, * *p* < 0.05, ** *p* < 0.01, *** *p* < 0.001.

Since neither CERK expression nor its product level explains why CERK inhibition specifically affects tamoxifen-resistant cells, we investigated the consequences of CERK inhibition on its substrate and product, i.e., ceramide and C1P levels in all three cell lines. We observed that NVP-231 treatment resulted in an increase in ceramides (Figure 4A,B), as well as dihydroceramides (Supplemental Figure S3), in all three cell lines. We next examined C16-C1P levels following CERK inhibition and found that NVP-231 treatment results in a significant decrease in C16-C1P levels in MCF-7 and MCF-7-HER2 cells and a trend towards lower C16-C1P levels in MCF-7-TAM1 cells (Figure 4C). It should be noted that the baseline level of C16-C1P was already low in MCF-7-TAM1 cells, which made detection of C1P in NVP-231-treated samples challenging. Overall, our data show that the consequences of CERK inhibition on its product and substrate are not different among tamoxifen-sensitive and -resistant cells, suggesting that differential CERK activity does not explain the increased sensitivity of tamoxifen-resistant cells to CERK inhibition.

#### 3.1.3. Tamoxifen-Resistant Cells Are More Sensitive to Ceramide-Induced Cell Death

**Figure 5:**
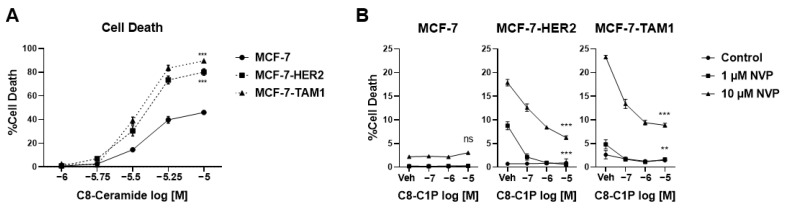
Tamoxifen-resistant cells are more sensitive to changes resulting from CERK inhibition. (**A**) MCF-7, MCF-7-HER2, and MCF-7-TAM1 cells were treated with increasing doses of C8-ceramide for 48-h and cell death was analyzed by Hoechst-PI staining. (**B**) C8-C1P was added to MCF-7, MCF-7-HER2, and MCF-7-TAM1 cells treated with NVP-231. After 48-h of treatment, cell death was measured by Hoechst-PI endpoint assay. ns: not significant, ** *p* < 0.01, *** *p* < 0.001.

We then asked whether tamoxifen-resistant cells have a different sensitivity to the consequences of CERK inhibition, i.e., an increase in ceramide and a decrease in C1P levels. Since ceramides are well-known inducers of apoptosis [54], we first investigated how tamoxifen-resistant and -sensitive cells respond to an increase in ceramides, as observed with NVP-231 treatment. To do this, we performed a dose-response analysis of C8-ceramide addition in the three cell lines and found that C8-ceramide induces significantly higher cell death in MCF-7-HER2 and MCF-7-TAM1 cells compared to MCF-7 parental cells, suggesting that the tamoxifen-resistant cells are more sensitive to increased ceramide levels compared to tamoxifen-sensitive cells (Figure 5A).

To investigate whether the product of CERK, C1P contributes to the differential sensitivity to CERK inhibition, we performed a lipid addback experiment in which cells were treated with NVP-231 and increasing concentrations of C8-C1P. We observed that C8-C1P partially reversed CERK inhibition-induced cell death in tamoxifen-resistant cells (Figure 5B) but had little to no effect in parental MCF-7 cells, suggesting that reduced C1P can also contribute to CERK inhibition-induced cell death.

**Figure 6:**
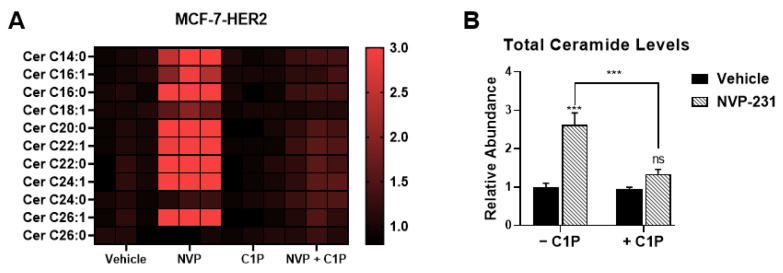
C1P addback partially reverses NVP-mediated ceramide increases. (**A**) MCF-7-HER2 cells were treated with 1 µM NVP-231, 1 µM C8-C1P, or a combination of NVP-231 and C8-C1P for 48-h. Abundance of all ceramide species relative to the vehicle-treated control was determined by LC-MS and is presented as a heatmap. (**B**) Total ceramide levels were calculated by dividing the abundance of all ceramide species by the average abundance of all ceramides in vehicle-treated controls and are presented as fold changes compared to vehicle-treated control in a bar plot. ns: not significant, *** *p* < 0.001.

Since C1P has been shown to downregulate ceramide levels by inhibiting the activity of serine palmitoyl transferase (SPT) [55], the rate-limiting enzyme for de novo ceramide biosynthesis [56], we examined how C1P addback affected ceramide levels in the MCF-7-HER2 cells. We found that C1P partially reverses the increase in ceramide levels induced by NVP-231 treatment (Figure 6A,B). Taken together, these findings support the concept that ET-resistant cells are more sensitive to CERK inhibition because they are more sensitive to ceramide-induced cell death and that one of the ways C1P promotes tamoxifen-resistant cell survival is by maintaining low endogenous ceramide levels.

## 4. Discussion

In this study, we analyzed the changes in the sphingolipidome associated with ET-resistant breast cancer. Our targeted analysis reveals that tamoxifen-resistant breast cancer cells have lower levels of ceramides and hexosylceramides compared to tamoxifen-sensitive cells. Follow-up studies revealed that tamoxifen-resistant cells are dependent on CERK, as it is required to maintain low ceramide levels necessary for tamoxifen-resistant cell survival. Mechanistically, C1P regulates de novo ceramide biosynthesis to promote survival. Consequently, the increase in ceramide levels upon CERK inhibition can be reversed in part by C1P, which acts as an important survival factor for tamoxifen-resistant cells.

Our findings raise an important question of how tamoxifen-resistant cells maintain a low level of ceramides. Typically, cells can employ multiple approaches to regulate the level of specific lipid species—regulate production via de novo synthesis or from salvage pathways or modulate its turnover. We observed that MCF-7-TAM1 cells have lower dihydroceramides; however, that was not consistent in MCF-7-HER2 cells. As dihydroceramides are upstream of ceramides, this suggests that ceramide biosynthesis may be decreased in some models of tamoxifen-resistance. Likewise, both tamoxifen-resistant cell lines demonstrate a potential increase in ceramide turnover but through different mechanisms. MCF-7-HER2 cells demonstrated increased levels of C1P and MCF-7-TAM1 cells showed increased sphingomyelins, suggesting that alterations in either biosynthesis or turnover of ceramides may be contributing to reduced endogenous ceramides in tamoxifen-resistant cells but that each cell line may utilize different mechanisms to achieve this outcome.

In addition to lower ceramides, we also observed that glucosylceramides were low in tamoxifen-resistant cell lines, indicated by the reduction of hexosylceramide species. Because glucosylceramides are downstream of ceramides, their reduced levels are more likely to be a consequence rather than a cause of reduced ceramide levels. Apart from being integral components for cell membranes, both ceramides and glucosylceramides play critical roles in cell signaling and gene expression [57]. Glucosylceramides can participate in the formation of lipid rafts, or glycosphingolipid-enriched microdomains in the plasma membrane and, therefore, can modulate several cellular signaling cascades [58,59]. Evidence from the literature suggests that increased ceramide glycosylation is one of the drivers of multidrug resistance in different cancers, through its ability to modulate drug transport and promote cellular signaling pathways aiding drug resistance [60,61]. However, as our results indicate that glucosylceramides levels are low in tamoxifen-resistant cells, tamoxifen resistance is likely not conferred by glucosylceramides, unlike other drug-resistant cancer cells.

Our data suggest that tamoxifen-resistant cells need to maintain low ceramide levels because they are more sensitive to ceramide-induced cell death. Ceramides are well-known mediators of apoptosis. Both short and long-chain ceramides form large channels in lipid bilayers in vitro [62,63], and similar ceramide channels also form in the outer mitochondrial membrane inducing cytochrome c release and an altered mitochondrial redox state [64]. Consequences of endogenous ceramide build-up include increased ROS [65], altered Ca^2+^ homeostasis [66,67], ATP depletion [68], and release of intermembrane space proteins [63]—all leading to apoptosis. Interestingly, a few recent reports have also described that ceramides can induce mitophagy resulting in cancer cell death [69,70]. However, the mechanisms of why tamoxifen-resistant cells have an increased sensitivity to ceramide-induced cell death remain to be elucidated. There are several reports which have described pro-apoptotic changes by ceramides could be reversed by overexpressing anti-apoptotic Bcl2 family genes [71,72,73]. Bcl2 family proteins are under the regulation of MAPK pathways [74] and some studies have reported increased MAPK signaling in endocrine therapy resistance, especially in long-term estrogen deprived models [75,76]. Therefore, it is possible that specific proteomic alterations in tamoxifen resistance contribute to increased sensitivity to ceramides, such as changes in the activity of Bcl2-family proteins, which warrants further investigation. A recent study using a photoactivable and clickable ceramide probe in transformed fibroblasts revealed that ceramide physically interacts with several endoplasmic reticula and mitochondrial proteins, including TRAM1, ERGIC3, and ANKLE2, to disrupt normal protein folding and respiratory functions [77]. However, the roles of these proteins in ET resistance are not well understood.

While tamoxifen-resistant models showed an increased sensitivity to CERK inhibition, CERK expression was not consistently different among the cell lines. This is in agreement with publicly available data showing that CERK mRNA levels in ER+ tumors are not associated with patient survival or responsiveness to tamoxifen [78,79]. Interestingly, some studies suggested that high CERK expression is correlated with HER2 status in breast cancer tumors [79,80]; however, none of these studies stratified patient outcome by ER status in HER2+ tumors. Of note, our HER2-overexpressed MCF-7 cell line did not have higher CERK mRNA or protein expression compared to the parental MCF-7 cell line. Similarly, CERK activity did not appear to be different between tamoxifen-sensitive and resistant cells, as there was no consistent difference in basal C1P levels between -sensitive and -resistant cells, and all cell lines demonstrated an increase in ceramides and a reduction in C1P in response to CERK inhibition. Interestingly, C1P has been shown to inhibit de novo ceramide synthesis in alveolar macrophages by inhibiting SPT, albeit specific interactions are not known [55]. Of note, we observed an increase in dihydroceramides in all cell lines upon CERK inhibition, suggesting an increase in de novo ceramide synthesis, which is consistent with C1P being a negative regulator of de novo ceramide biosynthesis. The addback of C1P could partially reverse CERK inhibition-induced cell death, as well as reduce ceramide levels. This suggests C1P is an important survival factor for tamoxifen-resistant cells as it can keep ceramide levels low likely by inhibition of de novo ceramide biosynthesis. In conjunction with its ability to reduce ceramides, C1P may play a survival role through other mechanisms such as activation of cytosolic phospholipase A2 [81], mTORC1 [82], PKCα [83], ERK1/2 [84], and JNK [84] as reported by several studies. However, these functions of C1P remain to be elucidated in ET-resistant breast cancer.

Taken together, our findings suggest that the ability of ET-resistant breast cancer cells to maintain a unique sphingolipid profile with low levels of ceramides is necessary for their survival. By targeting CERK and reducing the level of C1P, it is possible to exploit the sphingolipid profile of tamoxifen-resistant breast cancer by inducing ceramide-dependent cell death. Therapeutically, targeting CERK can therefore be a potential treatment modality in women with ER+ breast cancer that develop resistance to standard ETs.

## 5. Conclusions

To our knowledge, the work presented here represents the first lipidomic study of sphingolipids in ET-resistant breast cancer and led to our novel finding that ET-resistant breast cancer cells are more sensitive to ceramide-mediated cell death compared to ET-sensitive breast cancer cells. Moreover, our finding that ET-resistant cancer cells are dependent on CERK, and its product C1P, to maintain low levels of ceramides, and promote cell survival, indicates a new therapeutic target for ET-resistant breast cancer with the potential to improve outcomes for women with ET-resistant disease.

## Data Availability

Data is contained within the article or Supplementary Material.

## Supplementary Materials

The following supporting information can be downloaded at: https://www.mdpi.com/article/10.3390/cancers14102380/s1, Figure S1: NVP-231 dose response with or without 4-OHT in MCF-7-HER2 cells; Figure S2: Analysis of CERK expression in human breast tumors; Figure S3: NVP-231-induced alteration of the sphingolipidome in MCF-7, MCF-7-HER2, and MCF-7-TAM1 cells; Figure S4: The uncropped Western blots.
