# Supplementary material for: Endocrine Therapy-Resistant Breast Cancer Cells Are More Sensitive to Ceramide Kinase Inhibition and Elevated Ceramide Levels Than Therapy-Sensitive Breast Cancer Cells
Source: Cancers (Basel). 2022 May 12;14(10):2380. doi: 10.3390/cancers14102380 (PMC9140186; doi:10.3390/cancers14102380)
Supplement: Supplementary file 1 [file cancers-14-02380-s001.zip › cancers-1692122-supplementary.pdf]

cancers

Supplementary Material: Endocrine Therapy-Resistant Breast Cancer Cells Are More Sensitive to Ceramide Kinase Inhibition and Elevated Ceramide Levels than Therapy-Sensitive Breast Cancer Cells

Purab Pal, Alec Millner, Svetlana E. Semina, Rosemary J. Huggins, Logan Running, Diana S. Aga, Debra A. Tonetti, Rachel Schiff, Geoffrey L. Greene, G. Ekin Atilla-Gokcumen and Jonna Frasor

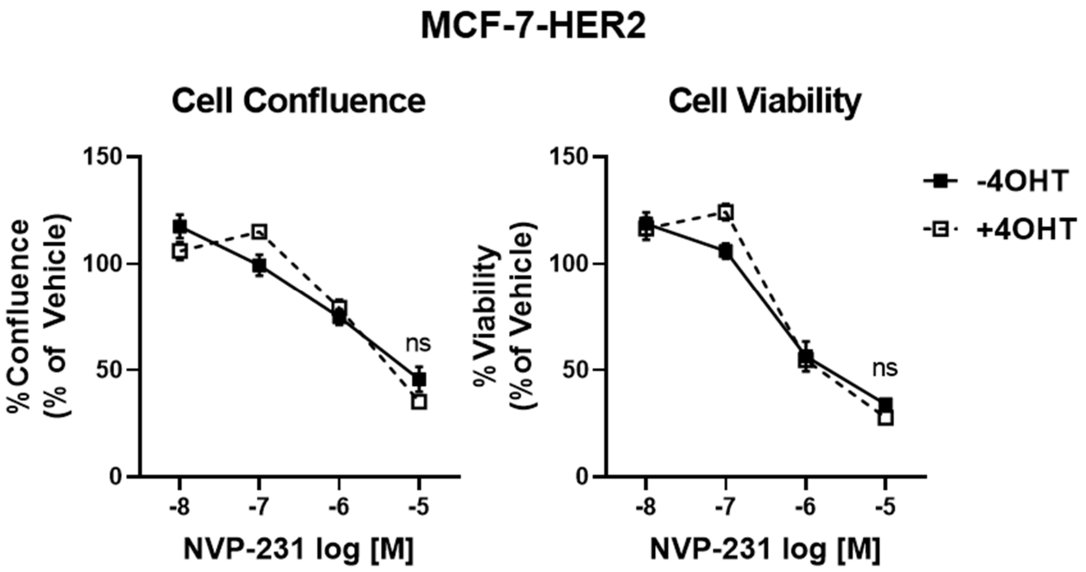

Figure S1. MCF-7-HER2 cells were treated with an increasing dose of NVP-231 with or without 1 μM of 4-hydroxytamoxifen (4-OHT). After 48-h of treatment, cell confluence (brightfield) and viability (PI-negative Hoechst-positive) were estimated from Hoechst-PI endpoint assay. ns: not significant.

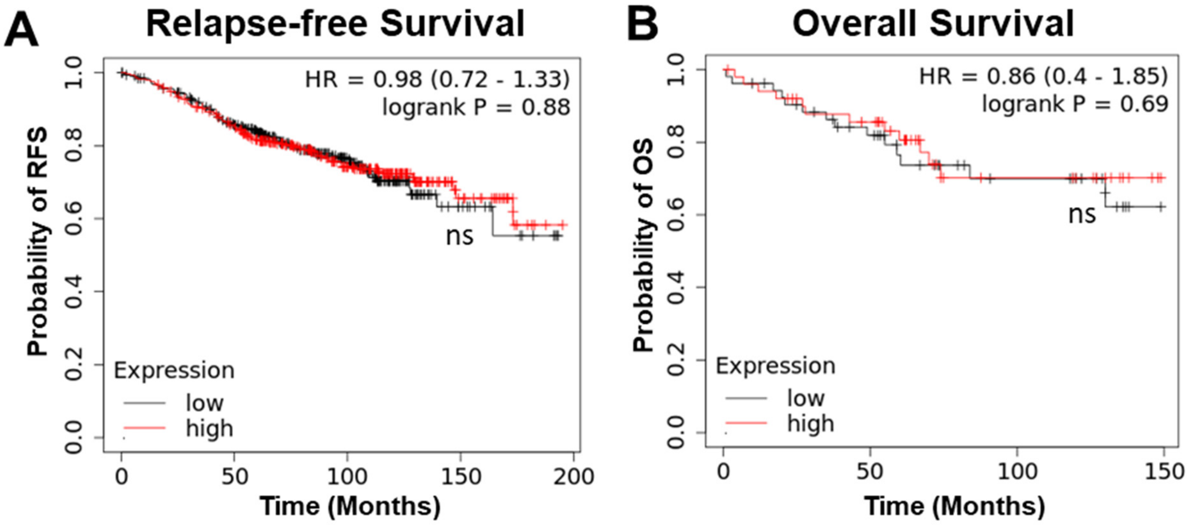

Figure S2. ER+ breast tumors treated with tamoxifen from publicly available datasets were analyzed for association between CERK mRNA expression and (**A**) relapse-free (n = 662) or (**B**) overall patient survival (n = 104). Statistical significance was determined using log-rank test. ns: not significant.

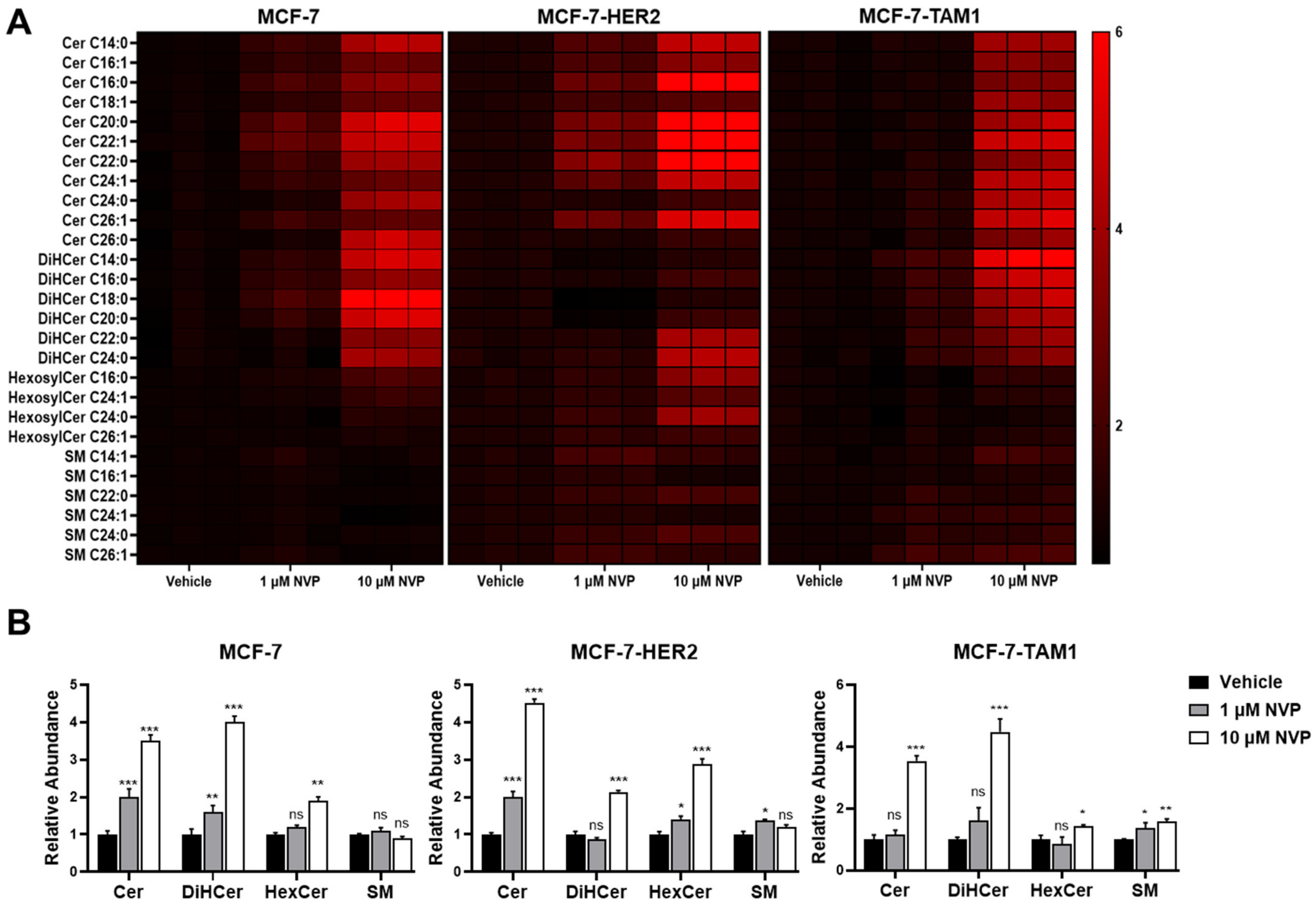

Figure S3. (**A**) MCF-7, MCF-7-HER2, MCF-7-TAM1 cells were treated with 1 and 10 μM of NVP-231 for 48-h and relative abundance of different sphingolipid species were measured relative to respective sphingolipid species levels in vehicle-treated (DMSO) control for each cell line and presented as heatmap. (**B**) Abundances of ceramides, dihydroceramides, hexosylceramides, and sphingomyelins in the NVP-231 treated groups relative to vehicle-treated controls were calculated by dividing the abundance of a certain sphingolipid species by the average abundance of that species in vehicle-treated controls for each cell line. ns: not significant, * $p < 0.05$, ** $p < 0.01$, *** $p < 0.001$.

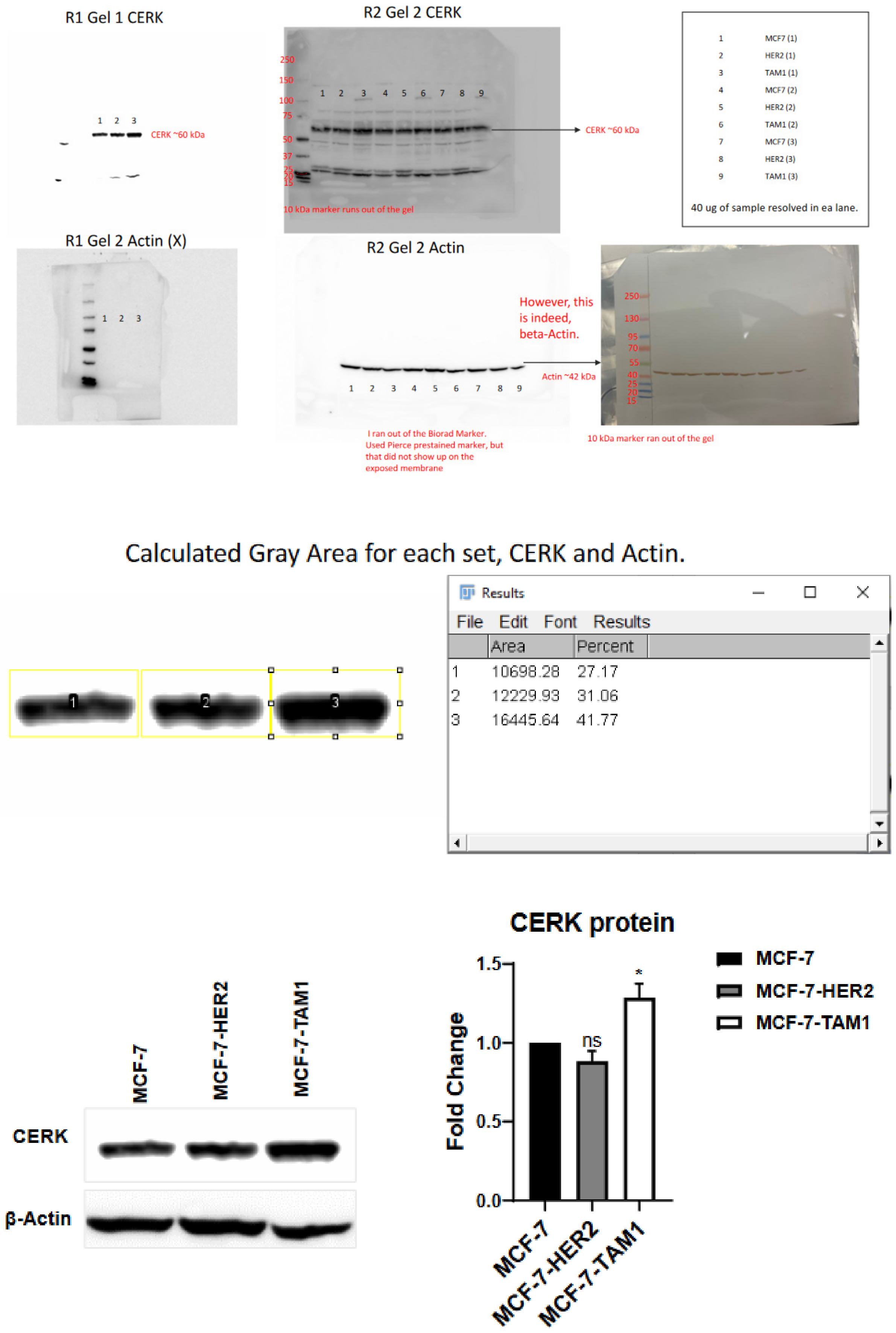

Figure S4. The uncropped Western blots.
